# Partial Deafness of Twenty Years Standing

**Published:** 1850-10

**Authors:** 


### ARTICLE XVIII.

### *Partial Deafness of Twenty Years Standing, Caused by the Presence of a Tooth Impacted in the Ear.*

The patient in this case, a shepherd, thirty-two years of age, applied to Dr. Niesmann for his aid in reference to deafness, from which he had suffered from childhood. He had been under Dr. Neismann's care two years previously, when suffering from an attack of typhus, at which time the doctor regarded the deafness as the result of cerebral disturbance, and which he considered he would lose with returning health.

On examining the organ with a sound, it struck against a hard bony substance; and on questioning the patient, he replied, "Yes, there is a tooth there." Dr. Neismann expressed his surprise at the answer, which was reiterated; and on further inquiry he learned that, when a boy at school, a school-fellow having removed a loose tooth for him, he jestingly inserted it into the external meatus, and it passed in beyond his reach. His hearing thereafter became impaired, and he was often quite deaf. Repeated but unavailing efforts had at various times been made for its extraction; he had suffered much pain, and lost much blood in these attempts, and had, in consequence of his deafness, been obliged to abandon his calling as a soldier, and had become a shepherd. Dr. Neismann introduced a pair of fine long forceps into the ear, and by a rotatory mot on extracted the tooth from the cavity of the tympanum. As if by an electric shock, the patient's previously dull and inexpressive countenance brightened up with joy, and he exclaimed, "Now I can hear." The tooth weighed five grains, and was coated with hard cerumen.—*Casper's Wochenschrift.*

